# An ATP7A‐Mediated Endogenous Regulation Bio‐Clay Enzyme Enables Self‐Amplified Copper Accumulation for Immunogenic Pyrop

**DOI:** 10.1002/advs.77973

**Published:** 2026-10-11

**Authors:** Weimin Xie, Yan Li, Guangyao Li, Ying Chen, Huaming Yang

**Affiliations:** ^1^ Engineering Research Center of Nano‐Geomaterials of Ministry of Education China University of Geosciences Wuhan China; ^2^ Faculty of Materials Science and Chemistry China University of Geosciences Wuhan China; ^3^ Laboratory of Advanced Mineral Materials China University of Geosciences Wuhan China

**Keywords:** ATP7A regulation, cascade catalytic reaction, immunogenic pyroptosis, montmorillonite, self‐amplification

## Abstract

Tumor cells maintain abnormal metabolism to support their rapid proliferation, which often compromises the therapeutic outcomes, including pyroptosis. Inhibiting the copper transporter ATPase (ATP7A) effectively disrupts this metabolic balance, yet current exogenous ATP7A inhibition strategies suffer from poor delivery efficiency and low tumor selectivity. Herein, we developed an endogenous regulation strategy to downregulate ATP7A levels using a self‐amplifying bio‐clay enzyme (Bio‐Clayzyme), cupriferous Prussian blue/montmorillonite (CuPB/Mt). Upon tumor cell internalization, CuPB/Mt induces a robust reactive oxygen species (ROS) storm, accompanied by antioxidant depletion, mitochondrial dysfunction, and adenosine triphosphate (ATP) exhaustion. These metabolic disturbances are associated with reduced ATP7A expression, which may subsequently impair ATP7A‐mediated copper efflux. The resulting intracellular copper accumulation may further promote ROS amplification, supporting a positive feedback loop among ROS production, ATP7A downregulation, and copper retention. Specifically, the introduction of Mt optimizes the Cu d‐band center and strengthens tumor cell adhesion, thereby boosting the multi‐enzyme‐like catalytic activity and enabling efficient utilization of short‐lived ROS. Consequently, CuPB/Mt activates nucleotide‐binding oligomerization domain‐like receptor family pyrin domain‐containing 3 (NLRP3)‐dependent pyroptosis and elicits a potent anti‐tumor immune response, offering an ATP7A‐mediated endogenous regulatory paradigm for self‐amplified catalytic therapy and immunogenic pyroptosis.

## Graphical abstract

A self‐amplifying bio‐clay nanozyme (Bio‐Clayzyme) disrupts tumor metabolic homeostasis through endogenous copper transporter ATPase (ATP7A) downregulation, enhancing cascade catalysis and triggering immunogenic pyroptosis for effective tumor immunotherapy.

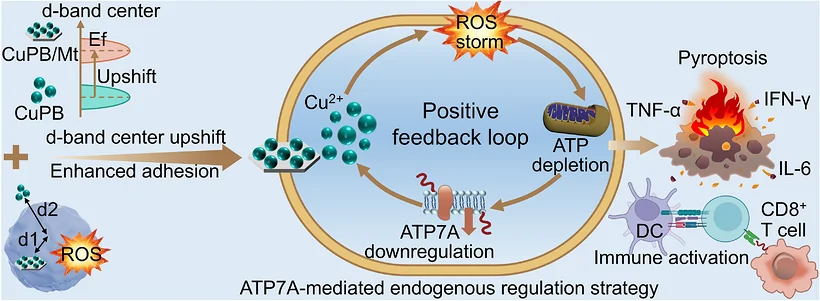

## 1 Introduction

Tumor immunotherapy has emerged as a transformative strategy for tumor treatment; however, its efficacy is frequently compromised by the immunosuppressive tumor microenvironment (TME) and the lack of sustained anti‐tumor immune activation [1, 2]. Pyroptosis, a lytic and highly inflammatory form of programmed cell death, has recently attracted considerable attention due to its ability to induce robust immunogenic cell death through the release of pro‐inflammatory cytokines and damage‐associated molecular patterns [3, 4, 5, 6]. Currently, extensive efforts have been devoted to triggering pyroptosis via reactive oxygen species (ROS)‐mediated disruption of intracellular redox homeostasis. Notably, the use of transition‐metal‐based nanomaterials for stimulus‐reactive anti‐tumor therapy has recently gained attention [7].

Among various transition metals [8, 9], copper stands out for its intrinsic biological relevance, versatile redox activity, and independence from external energy input, oxygen availability, or tissue penetration depth [10, 11, 12]. Remarkably, as a cofactor of redox‐active processes, copper‐based nanomaterials can deplete intracellular reductants, such as glutathione (GSH) and nicotinamide adenine dinucleotide (NADH), while catalyzing hydrogen peroxide (H_2_O_2_) into highly cytotoxic hydroxyl radicals (·OH) via Fenton‐like reactions [13, 14, 15]. This process effectively disrupts intracellular redox homeostasis and induces ROS‐dependent pyroptosis [16]. Recent studies have demonstrated that copper‐based nanoplatforms can amplify oxidative stress and synergistically activate pyroptosis‐associated pathways [17, 18]. Notably, compared with other transition metals (e.g., Pd, Pt, Ce, and Ru) [19, 20, 21], Cu exhibits superior biocompatibility as an essential trace element and maintains catalytic activity across a broader pH range [22, 23], enabling efficient Fenton‐like reactions under both mildly acidic and physiological conditions.

Despite these advances, the therapeutic efficacy of copper‐based nanomaterials is substantially limited by intrinsic cellular defense mechanisms. Specifically, copper transporter proteins, represented by copper transporter ATPase (ATP7A), can pump out excess intracellular copper ions, thereby maintaining copper homeostasis and attenuating copper‐induced pyroptosis [24, 25, 26, 27]. Moreover, compared with normal cells, ATP7A is frequently overexpressed in tumor cells, rendering them highly sensitive to ATP7A modulation [28]. Therefore, the inhibition of ATP7A expression represents an attractive strategy to disrupt copper homeostasis and enhance intracellular copper accumulation. However, current approaches predominantly rely on exogenous interventions, such as various ATP7A inhibitors (e.g., iodoacetamide [29]) and small interfering RNA [28], which suffer from poor delivery efficiency, limited tumor selectivity, and off‐target toxicity. In contrast, endogenous regulation strategies, which operate through in situ intracellular processes, offer greater targeted precision, stronger therapeutic specificity, and better biocompatibility while minimizing systemic side effects [30, 31]. Nevertheless, endogenous approaches for ATP7A modulation remain largely unexplored, and the mechanistic linkage between ATP7A suppression and pyroptosis activation has yet to be established. Therefore, developing a self‐sustaining and ATP7A‐mediated endogenous regulatory strategy represents a critical challenge in advancing next‐generation tumor immunotherapy.

In this study, we constructed a self‐amplifying Bio‐Clayzyme, a montmorillonite‐functionalized cupriferous Prussian blue nanoplatform (CuPB/Mt) to achieve ATP7A‐mediated endogenous regulation and amplify ROS production, thereby enhancing catalytic efficacy and triggering robust immunogenic pyroptosis (Scheme 1). Unlike representative Cu‐based nanozyme systems that primarily relied on defect engineering, single‐atom active‐site regulation, endogenous copper utilization, or exogenous enzyme/drug‐assisted metabolic intervention [11, 17, 18, 31], CuPB/Mt introduced a mineral‐attribute‐programmed strategy in which Mt served not merely as a passive support but as an active clay‐mineral regulator. Specifically, Mt modulated the electronic structure of Cu sites by upshifting the Cu d‐band center, thereby enhancing the peroxidase‐like (POD‐like), glutathione oxidase‐like (GSHOx‐like), and NADH oxidase‐like (NOx‐like) activities of CuPB/Mt. Meanwhile, the intrinsic tumor‐cell affinity of Mt promoted the cellular internalization and intracellular enrichment of CuPB/Mt, enabling proximity‐enhanced utilization of short‐lived ROS within intracellular compartments [32, 33, 34, 35]. The resulting intracellular ROS amplification disrupted redox homeostasis, induced mitochondrial dysfunction and ATP depletion, and was accompanied by reduced ATP7A expression and increased intracellular copper retention. This process established a self‐amplifying feedback loop that continuously enhanced ROS production and drove robust pyroptosis. As a proof of concept, both in vitro and in vivo studies demonstrated that CuPB/Mt effectively suppressed tumor growth and elicited systemic anti‐tumor immune protection. Collectively, this work presents a pioneering ATP7A‐mediated endogenous regulation strategy and highlights a general paradigm for self‐amplified catalytic therapy and pyroptosis‐based tumor immunotherapy.

**SCHEME 1:**
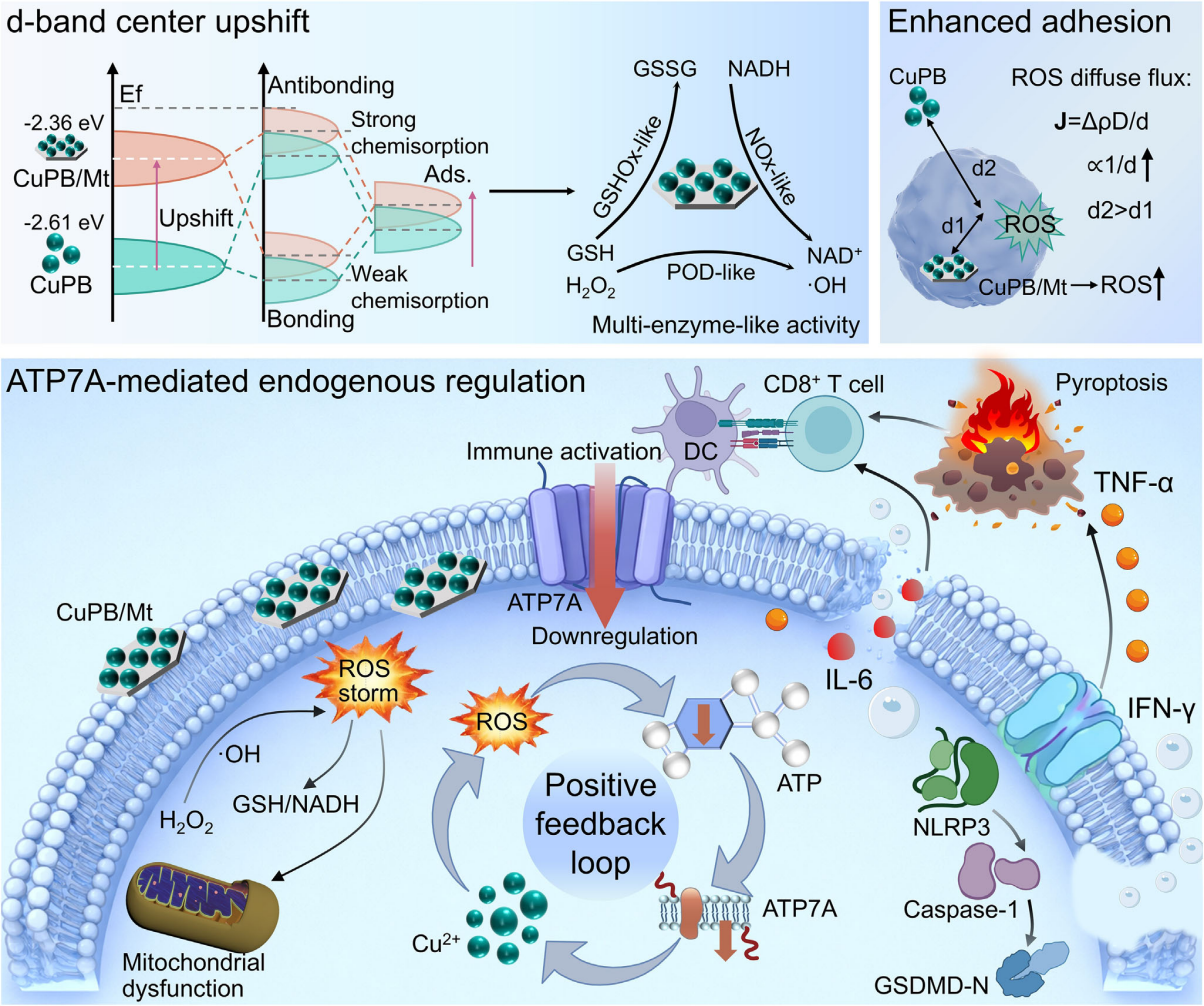
Schematic illustration of CuPB/Mt for activating anti‐tumor immunotherapy by inducing pyroptosis.

## 2 Results and Discussion

### 2.1 Fabrication and Characterization of CuPB/Mt

CuPB was prepared according to a reported method. A transmission electron microscopy (TEM) image showed that the prepared CuPB exhibited a typical coral‐like morphology, consisting of interconnected small nanoparticles approximately 30 nm in size (Figure 1a). Meanwhile, the corresponding TEM mapping images revealed a clear distribution of Cu and Fe elements (Figure S1). A CuPB/Mt nanozyme was synthesized using a simple one‐step solvothermal method, which resulted in the uniform distribution of CuPB on the lamellar surface of Mt (Figure 1b). Element mapping images revealed the presence of Al, Si, Cu, and Fe (Al and Si corresponding to Mt; Cu and Fe corresponding to CuPB), suggesting the successful anchoring of CuPB onto Mt (Figure 1c). X‐ray photoelectron spectroscopy (XPS) analysis of CuPB/Mt also confirmed the presence of distinct signals for Al, Si, Fe, and Cu (Figure S2), and inductively coupled plasma (ICP) analysis determined the percentages of CuPB and Mt in CuPB/Mt to be 59.5% and 40.5%, respectively (Figure S3). After loading, the hydrodynamic sizes of the Mt nanosheets increased and the zeta potentials varied from −7.4 to −11.9 mV (Figures S4 and S5).

**FIGURE 1:**
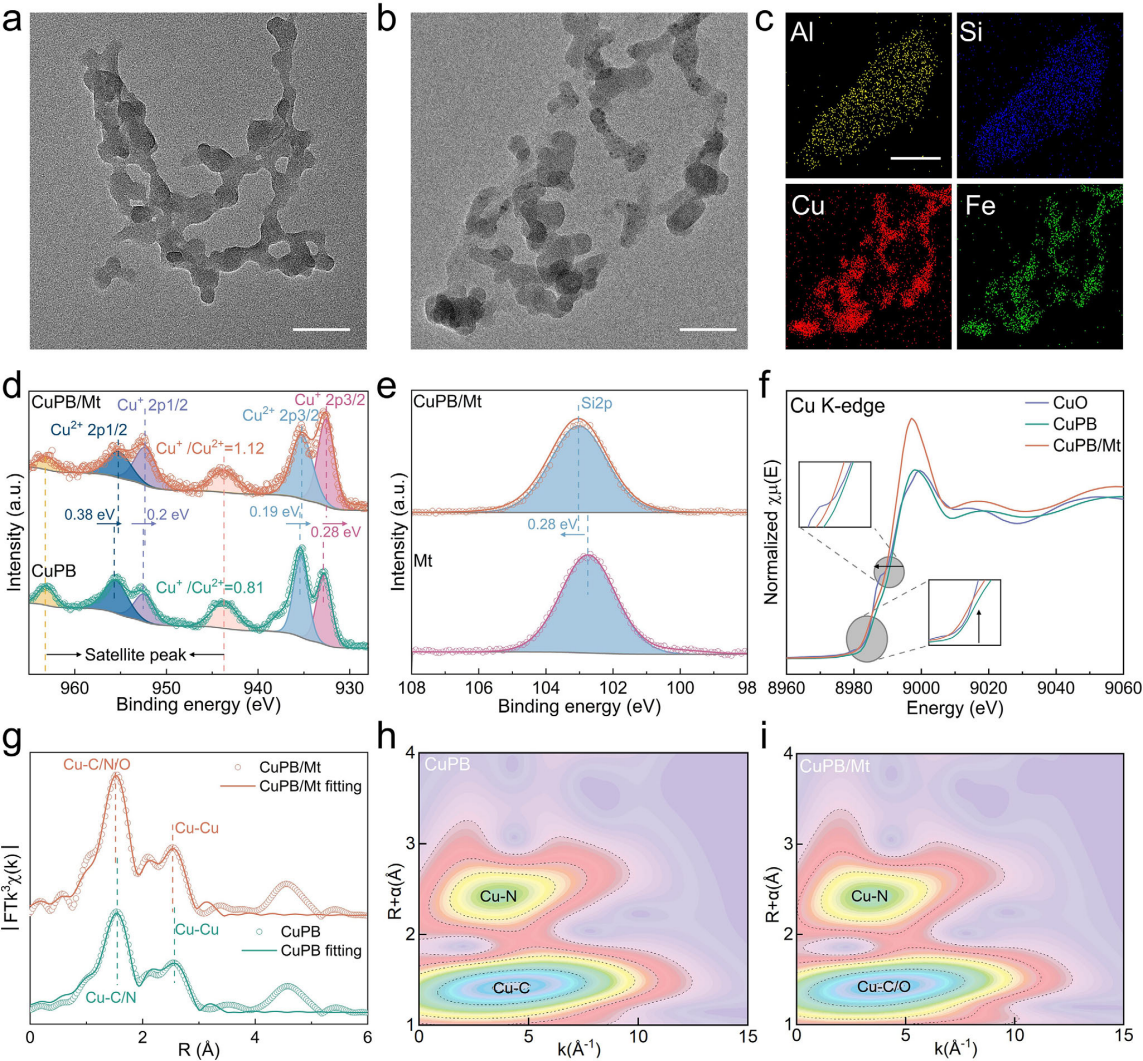
Characterizations of CuPB/Mt. TEM images of (a) CuPB and (b) CuPB/Mt (scale bar: 50 nm), (c) Corresponding element mapping images of CuPB/Mt (scale bar: 100 nm), XPS spectra of (d) Cu 2p for CuPB and CuPB/Mt and (e) Si 2p for Mt and CuPB/Mt, (f) XANES spectra of the normalized Cu K‐edge of CuPB and CuPB/Mt, (g) FT‐EXAFS curves of Cu, Wavelet transformations of (h) CuPB and (i) CuPB/Mt.

X‐ray powder diffraction (XRD) patterns showed the characteristic peaks of CuPB and Mt in CuPB/Mt, corresponding to Mt (PDF#13‐0135) and CuPB (PDF#75‐0022), respectively (Figure S6). Meanwhile, the Fourier transformed infrared (FTIR) spectrum of CuPB/Mt showed the characteristic peaks of CuPB and Mt (Figure S7), where the prominent peak at 2098 cm^−1^ was attributed to C≡N stretching vibrations from CuPB, and the peak at 1043 cm^−1^ was related to the Si─O planar vibrations from Mt. However, compared with CuPB or Mt alone, both peaks in CuPB/Mt were blueshifted, indicating the presence of interfacial interactions between CuPB and Mt. XPS spectra further revealed the chemical states of Cu and Si. The peaks of the high‐resolution Cu 2p spectrum for CuPB/Mt shifted slightly toward lower energy compared to those of CuPB (Figure 1d), while the peaks of the high‐resolution Si 2p spectrum were shifted toward higher energy compared to those of Mt (Figure 1e). The introduction of Mt increased the percentage of Cu^+^ in CuPB/Mt. These results suggested that interfacial charge transfer occurred between the two components and the charge was transferred from Mt to CuPB.

X‐ray absorption fine structure (XAFS) spectra were obtained to study the bonding states and coordination environment of CuPB/Mt. The Cu K‐edge of the X‐ray absorption near‐edge structure (XANES) spectra reflected the mean oxidation state of different Cu species, where the absorption edges of CuPB and CuPB/Mt were located close to CuO, showing that the valence state of Cu atoms was near Cu^2+^ (Figure 1f). Moreover, the mean oxidation state of the Cu in CuPB was slightly higher than that of the Cu in CuPB/Mt, suggesting a higher content of Cu^+^ in the latter. These results were consistent with the above XPS results (Figure 1d,e) and indicated that the transfer of electrons from Mt to CuPB in CuPB/Mt produced more Cu^+^ than that produced in CuPB alone. The extended XAFS (EXAFS) spectrum of CuPB displayed two dominant peaks at approximately 1.54 and 2.56 Å, corresponding to the Cu–C/N first and Cu–Cu second shells, respectively (Figure 1g and Figure S8). However, the first CuPB/Mt peaks became broadened, suggesting the presence of an additional Cu–O contribution at the CuPB/Mt interface. The corresponding structural parameters obtained from EXAFS curve fitting were summarized in Table S1. For CuPB/Mt, an additional Cu–O coordination shell was required to obtain a reliable fitting result, with a coordination number of 2.3 ± 0.4 and a bond distance of 1.91 ± 0.01 Å. This Cu–O distance was comparable to the Cu─O bond lengths in CuO and Cu_2_O references, but was much shorter than typical nonbonding physical adsorption distances, indicating the formation of interfacial Cu–O coordination rather than superficial physisorption. Meanwhile, the Cu–C/N coordination contribution was still retained in CuPB/Mt, suggesting that the original CuPB coordination framework was preserved. The formation of this interfacial Cu─O coordination could facilitate electron injection from the oxygen‐rich Mt surface to Cu sites in CuPB, thereby stabilizing a higher proportion of catalytically active Cu^+^ species. In addition, the wavelet‐transformed contour plots of CuPB and CuPB/Mt showed similar overall intensity distributions, further indicating that the CuPB framework was maintained after integration with Mt, while the local interfacial coordination environment was modulated by the additional Cu–O interaction (Figure 1h,i).

### 2.2 Evaluation of Multi‐Enzyme‐Mimetic Activities

The multi‐enzyme‐mimetic activities of CuPB/Mt were evaluated. Specifically, three enzymatic performances of CuPB/Mt, namely POD‐like, GSHOx‐like, and NOx‐like, were investigated in detail (the corresponding kinetic parameters are listed in Table S2). POD‐like activity, that is, the catalysis of H_2_O_2_ decomposition into ·OH, was first detected using H_2_O_2_ as a substrate and 3,3′,5,5′‐tetramethylbenzidine (TMB) as an indicator [36]. The ·OH produced by the POD‐like could catalyze the oxidation of TMB to blue‐colored oxidized TMB (oxTMB) with a characteristic absorption peak at 652 nm. Both CuPB and CuPB/Mt exhibited distinct characteristic absorption peaks at 652 nm, confirming their good POD‐like activity, while Mt showed almost no absorbance at 652 nm (Figure 2a and Figure S9). Furthermore, the absorption peaks of CuPB/Mt were notably stronger than those of CuPB, confirming its superior POD‐like activity. The TMB colorimetric reaction of different H_2_O_2_ concentrations satisfied typical Michaelis–Menten kinetics (Figure 2b). The maximum reaction rate (ν_max_) of CuPB/Mt was 5.75×10^−7^ m s^−1^, which is 1.47 times higher than that of CuPB (3.90×10^−7^ m s^−1^). Moreover, the catalytic constant (*K*cat, 2.65×10^−3^ s^−1^) and catalytic efficiency (*K*cat/*K*m, 6.07×10^−5^ mm ^−1^s^−1^) of CuPB/Mt were 1.56‐, 1.69‐fold higher than those of the CuPB (1.70×10^−3^ s^−1^ and 3.60×10^−5^ mm ^−1^s^−1^, respectively), suggesting a stronger ·OH generation capacity in CuPB/Mt compared to that in CuPB.

**FIGURE 2:**
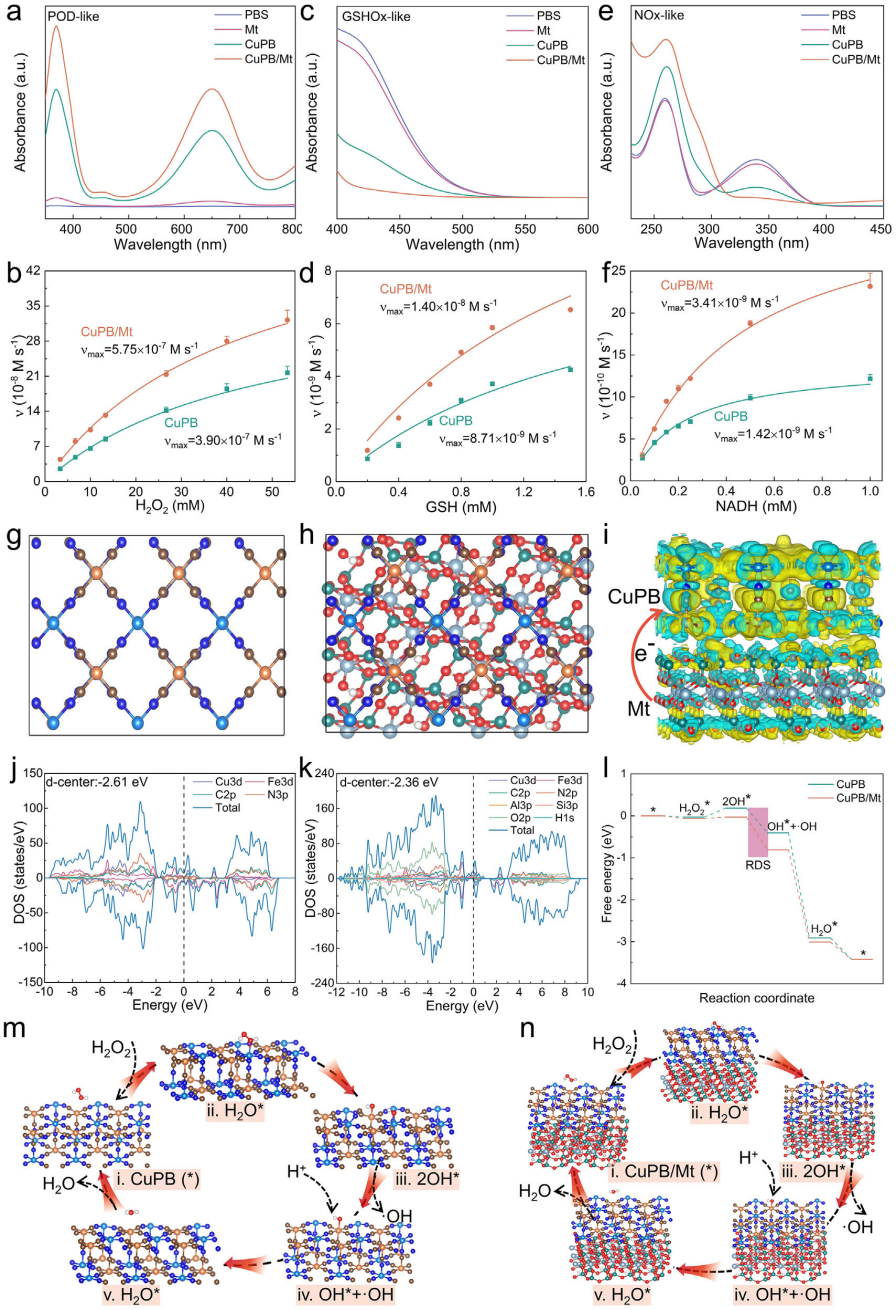
Multi‐enzyme‐mimetic activities and density functional theory calculations. (a) UV–vis absorption changes of H_2_O_2_ and TMB solution after different treatments, (b) POD‐like Michaelis–Menten kinetic analysis of CuPB and CuPB/Mt. (c) UV–vis absorption changes in GSH‐depletion detection after different treatments, (d) GSHOx‐like Michaelis–Menten kinetic analysis of CuPB and CuPB/Mt, (e) UV–vis absorption changes of NADH solution after different treatments, (f) NOx‐like Michaelis–Menten kinetic analysis of CuPB and CuPB/Mt. Optimized spatial configurations of (g) CuPB and (h) CuPB/Mt, (i) differential charge density of CuPB/Mt, density of states of (j) CuPB and (k) CuPB/Mt, (l) The corresponding free‐energy diagram for POD‐like reaction on CuPB and CuPB/Mt, proposed catalytic mechanism of (m) CuPB and (n) CuPB/Mt for POD‐like reaction.

Although CuPB/Mt demonstrated significant ROS‐generation capacity, the excessive GSH in TME could deplete the generated ROS, thus alleviating the oxidative stress in tumor cells and compromising the therapeutic effect of ROS‐based tumor therapy. Therefore, GSH depletion is an add‐on strategy for enhancing oxidative stress in tumor cells. Generally, GSHOx‐like activity leads to the oxidation of GSH into oxidized glutathione disulfide (GSSG), which consumes GSH and disrupts the redox balance of TME. In this study, the GSH‐depletion capacity was detected using 5,5′‐dithiobis‐(2‐nitrobenzoic acid) (DTNB), which reacted with GSH to produce yellow 2‐nitro‐5‐thiobenzoic acid (TNB) with a characteristic absorption peak at 412 nm [37]. As shown in Figure 2c and Figure S10, CuPB/Mt exhibited stronger GSH depletion ability than CuPB over a certain period of time, while Mt barely consumed GSH, confirming the considerably superior GSHOx‐like activity of the CuPB/Mt nanozyme. Meanwhile, the Michaelis–Menten equation representing the depletion of GSH in the CuPB and CuPB/Mt groups showed that CuPB/Mt exhibited a higher ν_max_ (1.40×10^−8^ m s^−1^) than that of CuPB (8.71×10^−9^ m s^−1^) (Figure 2d). In addition, CuPB/Mt exhibited 1.69 times higher *K*_cat_ (6.42×10^−5^ s^−1^) and 1.65 times higher catalytic efficiency (*K*_cat_/*K*_m_ = 4.23×10^−5^ mm ^−1^s^−1^) when oxidizing GSH, compared to those of CuPB (3.80×10^−5^ s^−1^ and 2.57×10^−5^ mm ^−1^s^−1^, respectively).

Even though CuPB can exacerbate ROS‐induced oxidative damage by depleting GSH, NADH, as a reductive coenzyme, can cooperate with intracellular GSH reductase to reduce GSSG back to GSH, thereby regenerating GSH. Fortunately, NOx‐like activity can oxidize NADH into NAD^+^, depleting NADH while further cascade‐magnifying oxidative stress by hindering GSH regeneration [38, 39]. Therefore, the rate of NADH consumption was determined by assessing the absorption peaks of NADH and NAD^+^ at 340 and 260 nm, respectively. After CuPB and CuPB/Mt treatment, the absorbance of NADH at 340 nm decreased, while the absorbance of NAD^+^ at 260 nm gradually increased, verifying the NOx‐like activity of CuPB and CuPB/Mt, while Mt had almost no NOx‐like activity, with relatively limited NADH depletion. CuPB/Mt demonstrated greater NADH‐consumption ability than CuPB (Figure 2e), and the reaction rates of different NADH concentrations conformed to typical Michaelis–Menten kinetics (Figure 2f). Meanwhile, CuPB/Mt exhibited a higher ν_max_ value (3.41×10^−9^ m s^−1^) toward NADH compared to CuPB (1.42×10^−9^ m s^−1^), and the *K*cat (1.56×10^−5^ s^−1^) and *K*cat/*K*m (3.64 ×10^−5^ mm ^−1^ s^−1^) of CuPB/Mt were 2.52 and 1.29 times higher than that of CuPB (6.20×10^−6^ s^−1^ and 2.82 ×10^−5^ mm ^−1^ s^−1^, respectively), which suggested that CuPB/Mt possessed more remarkable NOx‐like activity. Altogether, these results show that CuPB/Mt can more rapidly consume multiple reductive substances and produce more H_2_O_2_ than CuPB can, more toxic ·OH are generated through the cascade reactions, which is beneficial for follow‐up ROS‐based therapies.

### 2.3 Elucidation of the Catalytic Enhancement Mechanism of CuPB/Mt Nanozyme

To gain insights into the catalytic enhancement mechanism mediated by CuPB/Mt, density functional theory (DFT) calculations were performed. Optimized spatial configurations for CuPB and CuPB/Mt are illustrated in Figure 2g,h, while optimized spatial configurations for Mt are shown in Figure S11. The differential charge density was investigated based on the charge distribution in CuPB/Mt (Figure 2i), with yellow and green regions representing electron accumulation and dissipation, respectively. Electrons appeared depleted at Mt interfaces and aggregated at CuPB interfaces, indicating that electronic transfer occurred from Mt to CuPB at the interface. Combining the results of our previous study with the FTIR, XPS, and XANES results of the present study, the interfacial charge transfer channel was formed from Mt to CuPB. Furthermore, the density of states (DOS) distribution curves of CuPB/Mt showed a significant overlap between CuPB and Mt, revealing a strong interaction beneficial for accelerating electron transfer (Figure 2j,k). DOS analysis also indicated that the d‐band center (*E*d) of Cu 3d in CuPB/Mt (*E*d = −2.36 eV) was closer to the Fermi level than that in CuPB (*E*d = −2.61 eV). Furthermore, the upshift of *E*d values enhanced the adsorption capacity of CuPB/Mt for reaction intermediates and, as a result, boosted the enzyme‐mimicking reaction activity.

Taking POD‐like activity as an example, Figure 2l and Table S3 shows the Gibbs free energies of the POD‐like catalytic process, while the corresponding homogeneous cleavage steps of H_2_O_2_ catalyzed by CuPB and CuPB/Mt are presented in Figure 2m,n, respectively. H_2_O_2_ adsorption was the initial step (i), followed by conversion into activated H_2_O_2_^*^ (ii). The adsorption energy of the activated H_2_O_2_^*^ on CuPB/Mt (−0.06 eV) was lower than that on CuPB (−0.03 eV), indicating that CuPB/Mt had a stronger ability to adsorb H_2_O_2_^*^ molecules. Subsequently, each adsorbed H_2_O_2_ molecule was homogeneously cleaved into two ^*^OH groups (iii). One ^*^OH group was then dissociated and generated free ·OH (iv), which was the rate‐limiting step of the catalytic process. In this step, the energy barrier of CuPB/Mt (−0.78 eV) was lower than that of CuPB (−0.59 eV), indicating that the reaction was kinetically favorable and CuPB/Mt was more conducive to ·OH generation. Meanwhile, the other residual *OH was protonated to form H_2_O^*^ and, finally, desorbed to restore the catalyst to its original state (v). In summary, compared to CuPB alone, the introduction of Mt conferred strong interfacial electron transfer ability in CuPB/Mt and resulted in an increase in *Ed*. This effectively optimized the free energy of each step of the reaction and lowered the energy barrier for the formation of key intermediates, thereby accelerating the reaction process.

### 2.4 Disruption of Cellular Redox Homeostasis

Encouraged by the potent multi‐enzymatic activities of CuPB/Mt, the therapeutic effect and anti‐tumor mechanism were investigated at the cellular level. Initially, the endocytosis behaviors of Mt, CuPB, and CuPB/Mt in 4T1 cells were evaluated by labeling the materials with fluorescein isothiocyanate (FITC), a green fluorescent dye. The green fluorescence intensity of the CuPB/Mt‐treated group was higher than that of the CuPB‐treated group, indicating that the tumor cell adhesion of Mt enhanced the enrichment of CuPB/Mt in 4T1 cells (Figure S12). The cytotoxicity of CuPB/Mt against 4T1 cells was further evaluated using CellCounting Kit‐8 assays. Mt exhibited good biosafety, with the cell viability being close to 100% across the concentration range (Figure 3a), whereas CuPB and CuPB/Mt demonstrated concentration‐dependent cytotoxicity. Notably, compared with CuPB at the same concentration, CuPB/Mt had a stronger cell‐killing effect, with a cell survival rate of 23.58% at a concentration of 100 µg/mL. A flow‐cytometric assay of Annexin V‐FITC/PI staining showed that the apoptosis rate induced by CuPB/Mt was 51.4%, higher than those observed with Mt (3.68%) and CuPB (33.6%) alone (Figure 3b). Correspondingly, the results of live‐ and dead‐cell staining, performed using Calcein AM/PI, showed that cells treated with CuPB/Mt had the strongest red fluorescence and the weakest green fluorescence, suggesting that CuPB/Mt exhibited optimal 4T1 cell‐killing ability (Figure 3c).

**FIGURE 3:**
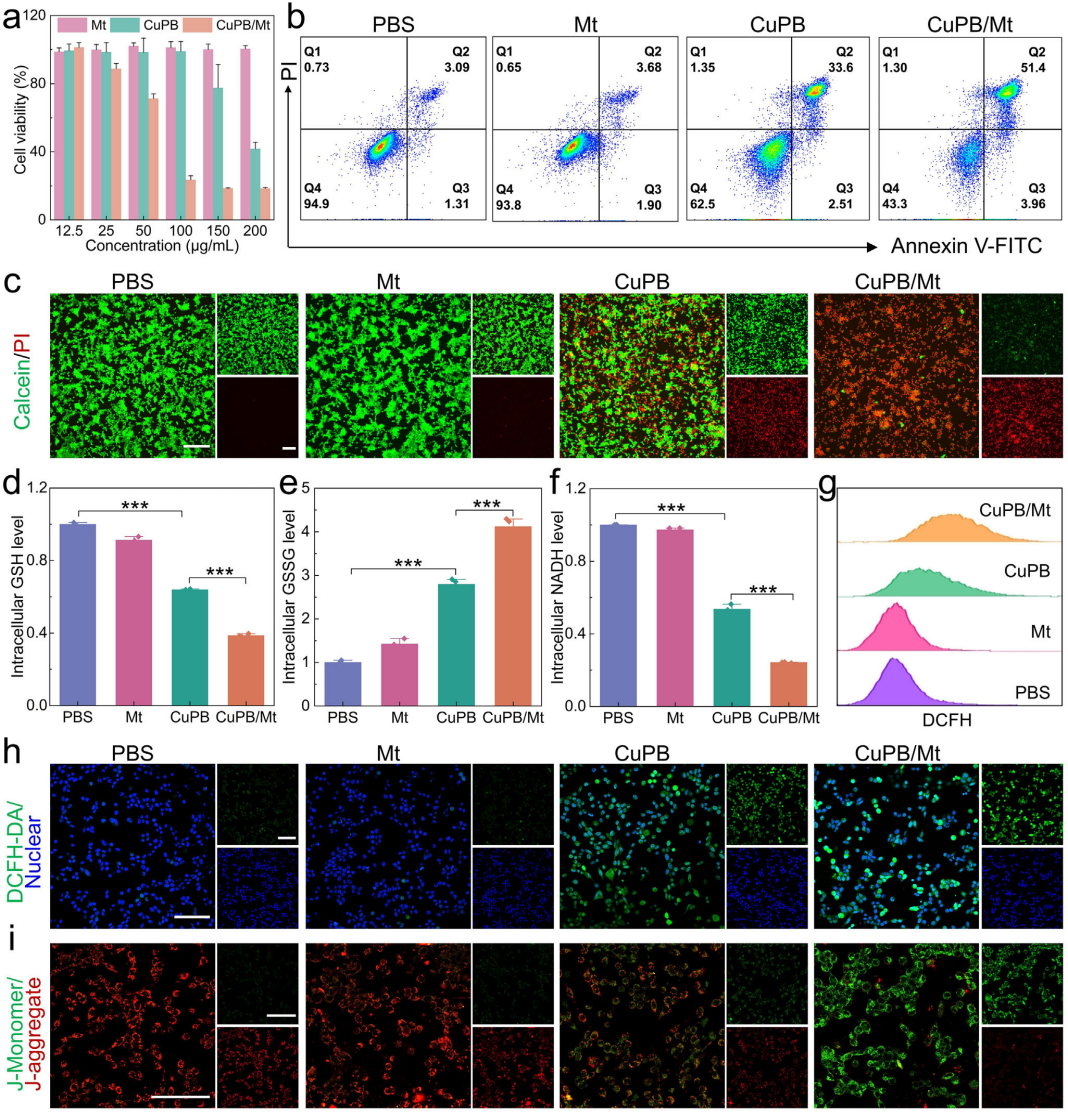
In vitro performance of CuPB/Mt. (a) Viability of 4T1 cells after different treatments, (b) Annexin V‐FITC/PI staining of 4T1 cells detected via flow cytometry, (c) Live/dead staining of 4T1 cells after different treatments (scale bar: 200 µm). Intracellular (d) GSH, (e) GSSG, and (f) NADH levels in different groups. Intracellular ROS levels stained using DCFH‐DA and detected using (g) flow cytometry and (h) confocal laser scanning microscopy (CLSM) images (scale bar: 100 µm), (i) Mitochondrial status detected using JC‐1 staining (scale bar: 100 µm). ^*^ *p* < 0.05; ^**^ *p* < 0.01; ^***^ *p* < 0.001.

Subsequently, fluctuations in intracellular redox homeostasis mediated by CuPB/Mt were monitored in the 4T1 tumor cell line. In addition, the GSH and GSSG levels were assessed using a commercial intracellular GSH and GSSG assay kit. The GSH level was lower in the CuPB group than in the PBS and Mt groups, suggesting inherent GSHOx‐like activity in CuPB (Figure 3d). The GSH level in the CuPB/Mt group was further reduced due to the enhanced biocatalytic activity. Correspondingly, the GSSG level was the highest in the CuPB/Mt group (Figure 3e). Given the pivotal role of NADH in maintaining redox homeostasis, intracellular NADH levels were measured using an NADH assay kit (Figure 3f). Compared with the other groups, the CuPB/Mt group exhibited the lowest NADH content, indicating the considerable disruption of intracellular redox homeostasis. These results showed that CuPB/Mt effectively limited the intracellular GSH levels by blocking the substrate and key enzymes involved in GSH synthesis and by depleting endogenous GSH.

Considering the POD‐like activity of CuPB/Mt and the depletion of intracellular GSH and NADH by CuPB/Mt, the intracellular ROS levels were detected using 2’,7’‐dichlorofluorescin diacetate (DCFH‐DA) staining. Compared with the other groups, the strongest green fluorescence was observed in the CuPB/Mt‐treated group, which indicated a pronounced disruption of intracellular redox homeostasis achieved through substantial GSH depletion and ROS generation (Figure 3g,h). The ROS storm produced by CuPB/Mt eventually caused irreversible oxidative damage and impaired mitochondrial function. As shown in Figure 3i and Figure S13, the CuPB/Mt‐treated group displayed the strongest green fluorescence (abnormal cells) and weakest red fluorescence (healthy cells), revealing severe mitochondrial damage.

### 2.5 Self‐Amplification of Intracellular Oxidative Stress via Endogenous Regulation

At the onset of mitochondrial dysfunction, the intracellular ATP content in the CuPB/Mt group was the lowest among all groups, indicating a substantial reduction in cellular energy supply for tumor proliferation (Figure 4a). This ATP depletion was accompanied by downregulated expression of the copper exporter ATP7A, which may impair ATP7A‐associated copper efflux and contribute to intracellular copper accumulation. As a proof of concept, Western blotting (WB) analysis was performed, and the results showed that the ATP7A level in the CuPB/Mt group was markedly lower than that in the PBS, Mt, and CuPB groups, while β‐actin remained comparable (Figure 4b). Correspondingly, the total intracellular copper level in the CuPB/Mt‐treated cells was significantly higher than that in cells treated with CuPB alone (Figure 4c), indicating efficient copper retention within tumor cells after CuPB/Mt treatment. The elevated intracellular copper level may further facilitate Cu‐based redox cycling and ROS generation, supporting a self‐amplifying feedback process involving intracellular copper accumulation, ROS production, mitochondrial dysfunction, ATP depletion, and ATP7A downregulation. Such feedback could sustain oxidative stress and contribute to tumor cell death.

**FIGURE 4:**
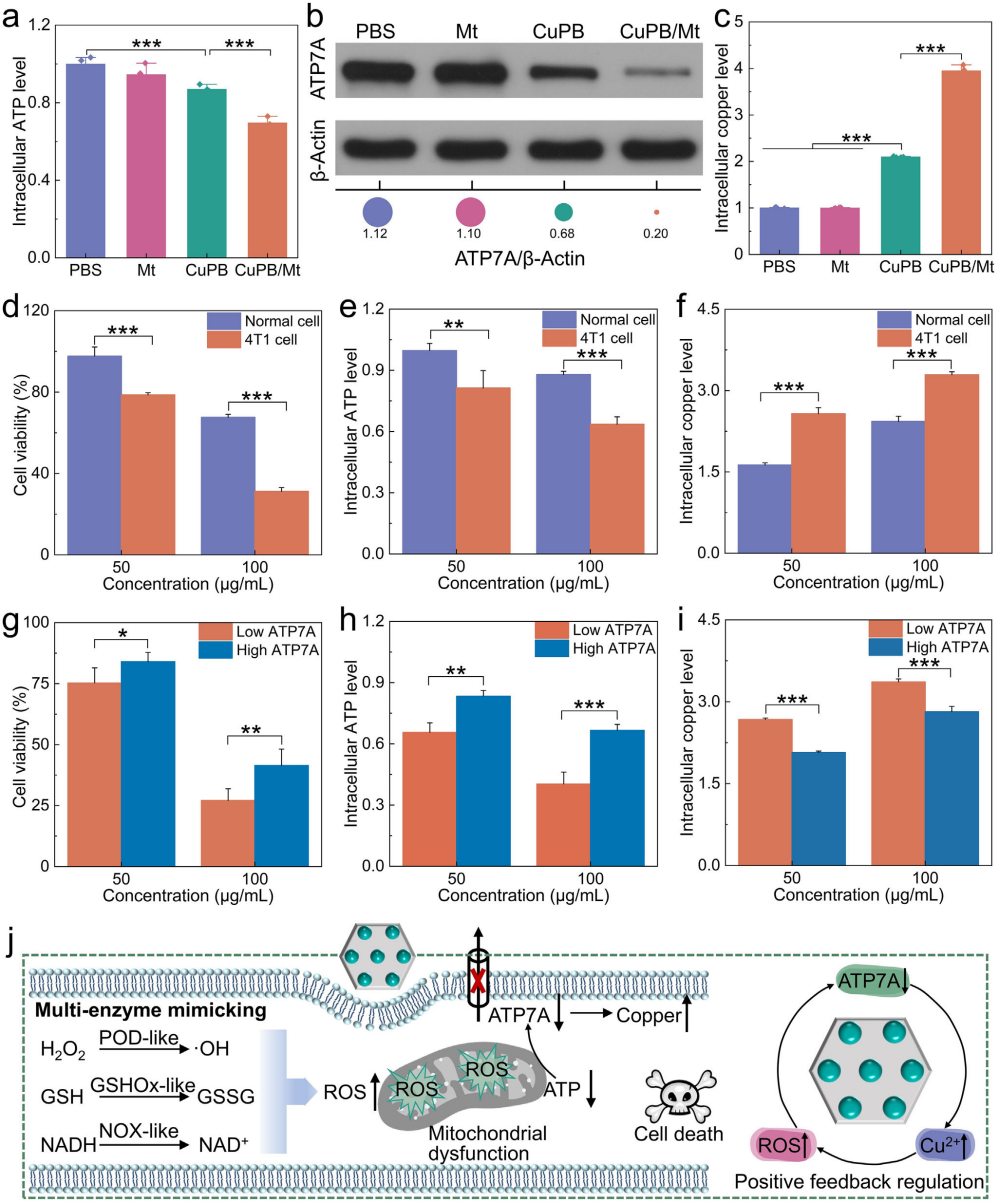
(a) Relative intracellular ATP levels after different treatments, (b) Western blot analysis of ATP7A expression after different treatments, (c) Relative intracellular copper levels after different treatments; (d) Cell viability, (e) relative intracellular ATP levels and (f) relative intracellular copper levels in normal cells and 4T1 cells after CuPB/Mt treatment; (g) Cell viability, (h) relative intracellular ATP levels and (i) relative intracellular copper levels in parental 4T1 cells and ATP7A‐upregulated 4T1 cells after CuPB/Mt treatment; (j) Schematic illustration of ATP7A‐mediated endogenous copper‐retention feedback induced by CuPB/Mt. ^*^ *p* < 0.05; ^**^ *p* < 0.01; ^***^ *p* < 0.001.

To assess whether CuPB/Mt perturbed the broader copper‐homeostasis network, representative copper transport and trafficking regulators, including ATP7B, CTR1, and ATOX1, were examined by WB. As shown in Figure S14, CuPB/Mt treatment markedly reduced the protein levels of ATP7B and ATOX1, which may compromise ATP‐dependent copper export and chaperone‐mediated copper trafficking. CTR1 expression was also reduced, potentially reflecting a compensatory response to intracellular copper accumulation that could limit further copper uptake. Nevertheless, this compensation was insufficient to prevent intracellular copper retention, likely because ATP7A/ATP7B‐mediated copper efflux and ATOX1‐mediated copper trafficking were simultaneously impaired. Furthermore, total cellular protein content was measured to assess whether the observed changes were accompanied by an overall reduction in protein content. Total protein levels remained comparable among the PBS, Mt, CuPB, and CuPB/Mt groups (Figure S15). This observation, together with the decreased expression of ATP7A and the other examined copper‐homeostasis regulators, suggests preferential perturbation of copper‐homeostasis‐related proteins, although broader stress‐response mechanisms cannot be completely excluded.

Previous studies have reported that ATP7A expression was significantly upregulated in breast invasive carcinoma compared to normal tissues [28], providing a rationale for investigating ATP7A‐associated copper‐homeostasis perturbation in breast tumor cells. Consistent with this, CuPB/Mt exhibited greater cytotoxicity toward 4T1 cells than toward the normal cells examined (Figure 4d), accompanied by a more pronounced decline in intracellular ATP levels and an increase in total intracellular copper levels (Figure 4e,f). These observations indicate greater susceptibility of 4T1 cells to CuPB/Mt under the tested conditions. To further investigate the functional contribution of ATP7A, an ATP7A‐upregulated 4T1 cell model was established (Figure S16). Following CuPB/Mt treatment, ATP7A‐upregulated cells exhibited significantly higher viability and intracellular ATP levels, together with lower total intracellular copper levels, than parental 4T1 cells, supporting the functional involvement of ATP7A in the observed cellular response (Figure 4g–i).

Collectively, the reduced expression of ATP7B, ATOX1, and CTR1 (Figure S14), together with ATP7A downregulation (Figure 4b), indicates perturbation of multiple components of the copper‐homeostasis network. The partial rescue observed in ATP7A‐upregulated cells (Figure 4g–i) further supports ATP7A as a major, but not exclusive, mediator of this response. These findings are consistent with a proposed feedback model in which intracellular copper accumulation and oxidative stress reinforce each other within the broader framework of copper‐homeostasis perturbation, potentially contributing to CuPB/Mt‐mediated disruption of tumor metabolic homeostasis and anti‐tumor activity (Figure 4j).

### 2.6 Activation of Pyroptosis‐Associated Immunogenic Response

Pyroptosis, a type of inflammatory programmed cell death, can be triggered by excessive intracellular ROS. Therefore, the immunogenic pyroptosis induced by CuPB/Mt was further explored. Given that cell swelling accompanied by large bubbles is a hallmark feature of pyroptosis, the cell morphology after different treatments was assessed using a microscope and Bio‐TEM. Compared with the PBS group, CuPB/Mt‐treated 4T1 cells exhibited obvious swelling and abundant bubble‐like structures (white arrows, Figure S17), suggesting pyroptosis‐like morphological changes. Consistently, Bio‐TEM images showed apparent membrane rupture (red arrows), extensive intracellular vacuolization (yellow arrows), and severe mitochondrial damage (white arrows) in the CuPB/Mt group, whereas CuPB alone caused only moderate membrane deformation and mitochondrial injury (Figure 5a). These results indicate that CuPB/Mt induced more severe cellular damage than CuPB, which was closely associated with its enhanced cascade enzymatic activity and intracellular ROS amplification.

**FIGURE 5:**
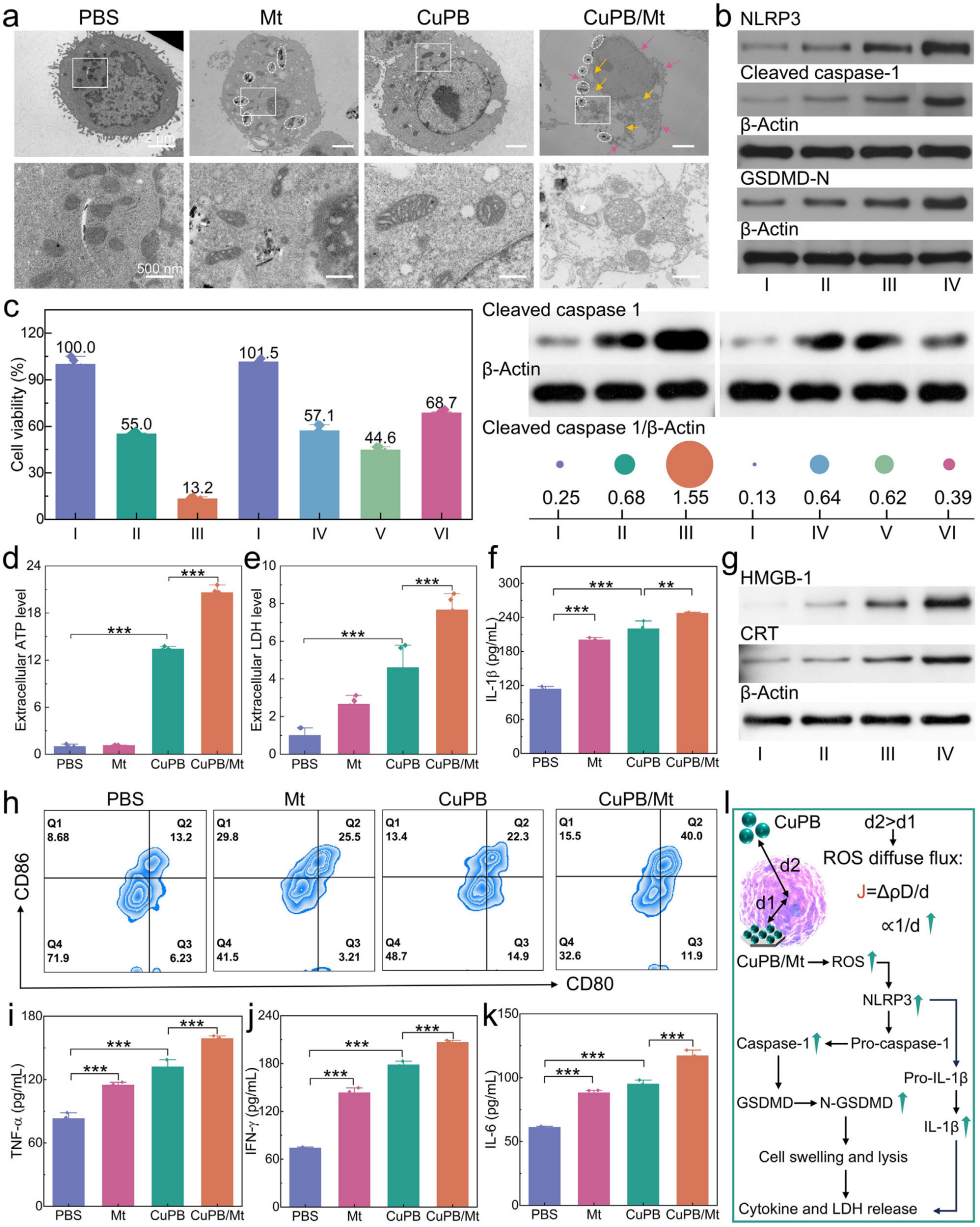
Immunogenic pyroptosis mediated by CuPB/Mt. (a) Bio‐TEM images of 4T1 cells after different treatments (scale bar: above, 2 µm; below, 500 nm), (b) Western blot analysis of NLRP3, cleaved caspase‐1 and GSDMD‐N after different treatments (I: PBS, II: Mt, III: CuPB, and IV: CuPB/Mt), (c) Cell viability and western blot assays of 4T1 cells after different treatments (I: PBS, II: CuPB/Mt+NAC, III: CuPB/Mt, IV: CuPB/Mt+Z‐VAD‐FMK, V: CuPB/Mt+UK‐5099, and VI: CuPB/Mt+VX‐765), Secretion of (d) ATP, (e) LDH and (f) IL‐1𝛽 after different treatments, (g) Western blot analysis of HMGB1 and CRT after different treatments (I: PBS; II: Mt; III: CuPB; IV: CuPB/Mt), (h) Representative flow cytometry plots of DCs, secretion levels of (i) TNF‐𝛼, (j) IFN‐γ and (k) IL‐6 in the supernatant of DCs after different treatments, (l) Schematic illustration of the possible mechanism of action of CuPB/Mt in promoting immunogenic pyroptosis. ^*^ *p* < 0.05; ^**^ *p* < 0.01; ^***^ *p* < 0.001.

Apart from its catalytic activity, CuPB/Mt also benefited from favorable interfacial interactions with tumor cells. Bio‐TEM images revealed that abundant nanoparticles adhered to or were internalized by 4T1 cells in the Mt and CuPB/Mt groups (white circles, Figure 5a). This suggested that Mt facilitated the cell internalization and intracellular enrichment of CuPB/Mt, thereby enhancing intracellular proximity‐dependent ROS‐mediated oxidative damage. In contrast, CuPB alone exhibited minimal cellular adhesion, likely due to its smooth surface and limited interaction with tumor cells.

Subsequently, to elucidate the pyroptosis‐related molecular mechanism, the expression of representative pyroptosis biomarkers was examined. In the classical pyroptosis pathway, the NOD‐like receptor protein 3 (NLRP3) inflammasome can activatecaspase‐1, which further cleaves a downstream effector protein, gasdermin‐D (GSDMD), to release its N‐terminal structural domain (GSDMD‐N), ultimately inducing membrane pore formation and inflammatory cell death [40, 41]. WB analysis showed that CuPB treatment upregulated NLRP3, cleaved caspase‐1, and GSDMD‐N compared with PBS and Mt, indicating the activation of pyroptosis‐related signaling (Figure 5b). Notably, CuPB/Mt further increased the levels of these proteins, demonstrating its stronger ability to activate the NLRP3/caspase‐1/GSDMD pathway.

To verify the role of ROS and pyroptosis in CuPB/Mt‐induced cell death, inhibitor experiments were further performed. Scavenging intracellular ROS with N‐acetylcysteine (NAC) [42, 43] markedly restored cell viability and reduced cleaved caspase‐1 expression, confirming that ROS amplification was a critical upstream trigger of pyroptosis (Figure 5c). Similar rescue effects were observed when CuPB/Mt was co‐incubated with representative inhibitors of apoptosis, cuproptosis, and pyroptosis, including Z‐VAD‐FMK [44], UK‐5099 [45], and VX‐765 [46, 47], respectively. Among these inhibitors, VX‐765 resulted in the highest cell viability and the lowest cleaved caspase‐1 levels, suggesting that pyroptosis appeared to be a major contributor to CuPB/Mt‐induced cell death (Figure 5c).

Pyroptotic membrane perforation is generally accompanied by the release of intracellular contents and inflammatory mediators. Therefore, extracellular ATP, lactate dehydrogenase (LDH), and interleukin‐1β (IL‐1β) levels in the culture supernatants were quantified. CuPB/Mt treatment induced the highest extracellular ATP, LDH, and IL‐1β levels among all groups, which were 20.6‐, 7.67‐, and 2.17‐fold higher than those in the PBS group, respectively (Figure 5d–f). Notably, the decrease in intracellular ATP level (Figure 4a) and the increase in extracellular ATP levels (Figure 5d) reflected different aspects of the cellular response to CuPB/Mt. Intracellular ATP depletion was associated with mitochondrial dysfunction and impaired energy metabolism, whereas elevated extracellular ATP was consistent with the release of intracellular contents during pyroptosis‐associated membrane permeabilization. To further assess immunogenic activation, danger signals associated with immunogenic cell death (ICD) were examined. CuPB/Mt treatment markedly increased the cellular protein levels of high‐mobility group box 1 (HMGB1) and calreticulin (CRT) compared with those in the PBS, Mt, and CuPB groups (Figure 5g). Together with the observed increases in NLRP3, cleaved caspase‐1, and GSDMD‐N levels, as well as extracellular ATP release, LDH leakage, and IL‐1β secretion, these findings support pyroptosis‐associated immunogenic activation following CuPB/Mt treatment.

### 2.7 In Vitro Immune Responses Mediated by CuPB/Mt

Given that CuPB/Mt‐induced pyroptotic cell death was accompanied by the release of inflammatory mediators and ICD‐associated danger signals, its potential to stimulate anti‐tumor immune responses was next evaluated in vitro. Generally, dendritic cells (DCs) play a central role in the activation and polarization of T cells. Therefore, bone marrow‐derived DCs (BMDCs) were co‐incubated with differently treated 4T1 cells, and DC maturation was evaluated by quantifying the CD80^+^CD86^+^ population. As shown in Figure 5h, CuPB/Mt treatment induced the highest proportion of mature DCs, with the CD80^+^CD86^+^ population reaching 40.0%, compared with only 13.2% in the PBS group. Notably, Mt treatment also increased the DC maturation rate to 25.5%, suggesting a moderate immunoadjuvant‐like effect. This effect may arise from the intrinsic physicochemical properties of Mt, including its hydroxyl‐rich layered surface, exchangeable interlayer cations, and its interfacial interactions with cells [32, 42], which may facilitate antigen adsorption and subsequent antigen presentation by DCs. Furthermore, enzyme‐linked immunosorbent assays (ELISA) were conducted to evaluate the secretion of immune‐related cytokines during DC maturation. Compared with the other groups, CuPB/Mt treatment significantly increased the secretion levels of tumor necrosis factor‐α (TNF‐α), interferon‐γ (IFN‐γ), and interleukin‐6 (IL‐6), indicating that CuPB/Mt effectively promoted DC activation and cytokine secretion (Figure 5i–k).

Collectively, CuPB/Mt combined enhanced cascade catalytic activity with Mt‐mediated cellular association and intracellular enrichment, thereby amplifying intracellular ROS production and disrupting cellular homeostasis. The resulting oxidative stress activated the NLRP3/caspase‐1/GSDMD pathway, promoted pyroptosis‐related membrane permeabilization, and increased extracellular ATP, LDH, and IL‐1β levels. These changes were further accompanied by elevated cellular levels of the ICD markers HMGB1 and CRT. Together with enhanced DC maturation and cytokine secretion, these findings support a role for ROS‐driven pyroptosis in CuPB/Mt‐induced immunogenic activation (Figure 5l).

### 2.8 Evaluation of in Vivo Anti‐Tumor Effects

Considering the remarkable tumor cell killing and the pyroptosis‐associated immunogenic activation in vitro, the anti‐tumor properties of CuPB/Mt were investigated in vivo. 4T1 tumor‐bearing BALB/c mice were randomly divided into four groups and intravenously injected with PBS, Mt, CuPB, and CuPB/Mt separately on days 0, 2, and 4. The body weights and tumor sizes of the mice were monitored during the treatments. The therapeutic effects are shown in Figure 6a,b. Tumors grew unrestrictedly in both the PBS and Mt groups, whereas the tumor growth in the CuPB group was slightly controlled, indicating a modest tumor‐suppressive effects. By contrast, CuPB/Mt significantly inhibited tumor proliferation, consistent with the in vitro cytotoxicity results. Meanwhile, the body weights of the mice remained relatively stable, indicating good biosafety of the treatment (Figure S18). At the end of therapy, the strong tumor‐inhibition efficacy of CuPB/Mt was further confirmed by weighing the resected tumors, and the tumor inhibition rate was 82.8% (Figure 6c and Figure S19). No obvious abnormalities were observed in major‐organ histology (Figure S20), blood routine (Figure S21), or blood biochemical (Figure S22), indicating the good biocompatibility and biosafety of these treatment strategies.

**FIGURE 6:**
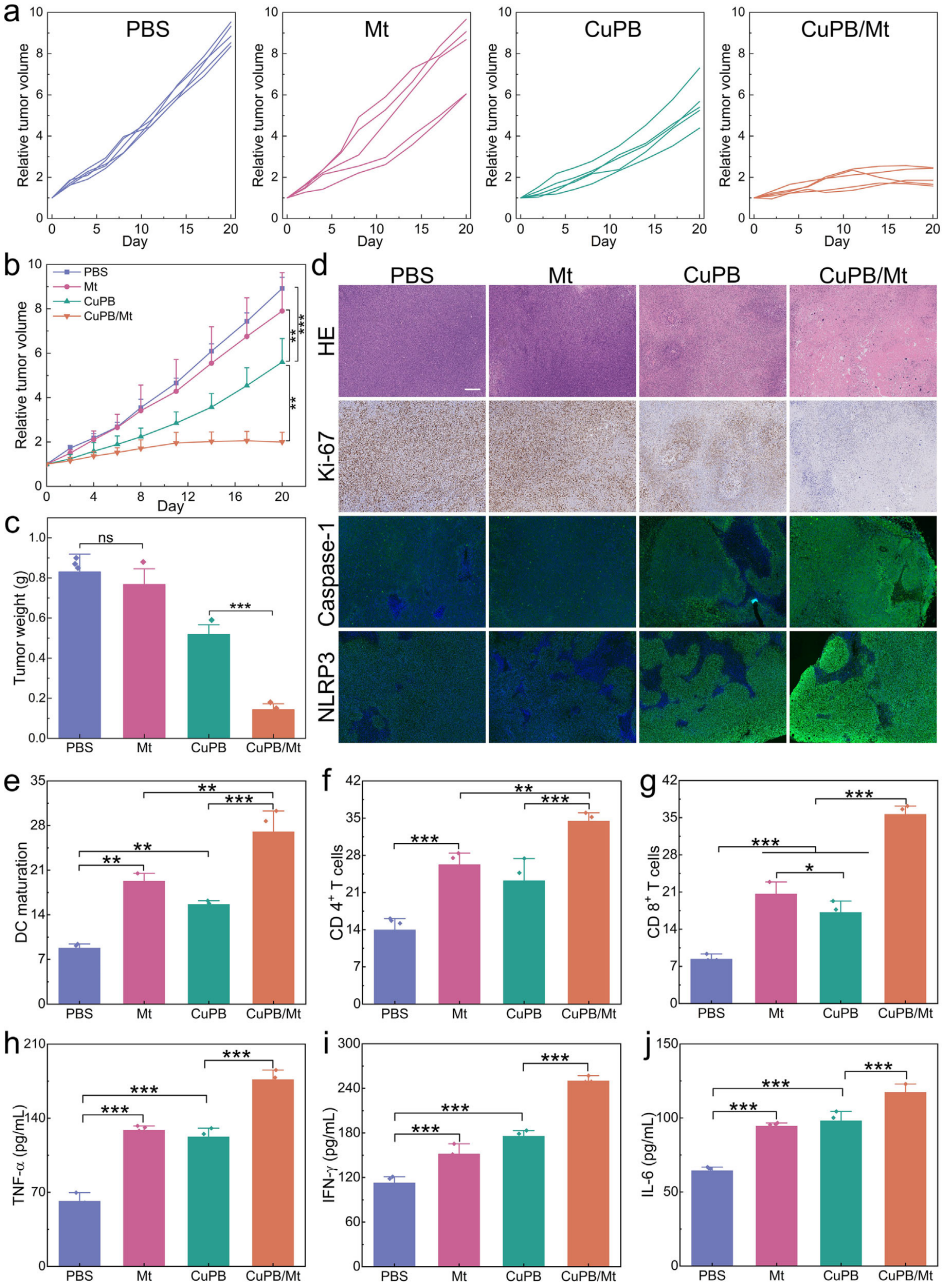
In vivo anti‐tumor effects and immune activation. (a) Individual tumor growth curves and (b) average tumor growth curves after different treatments, (c) Tumor weight at the end of therapy, (d) Tumor sections stained by H&E, Ki‐67, cleaved caspase‐1, and NLRP3 after different treatments (scale bar, 200 µm). Flow cytometry quantitative analysis of (e) mature DCs, (f) CD3^+^ CD4^+^ T cells, and (g) CD3^+^ CD8^+^ T cells in the tumors. The secretion levels of (h) TNF‐α, (i) IFN‐γ, and (j) IL‐6 in the serum of mice after different treatments. ^*^ *p* < 0.05; ^**^ *p* < 0.01; ^***^ *p* < 0.001.

Additionally, a histological evaluation of the resected tumor tissues was performed using hematoxylin and eosin (H&E) and Ki‐67 staining to assess the therapeutic effects. As illustrated in Figure 6d, the CuPB/Mt‐treated group showed the most severe destruction of tumor cells, based on H&E staining, as well as the most significant suppression of tumor cell proliferation, as determined using the Ki‐67 assay, indicating its pronounced anti‐tumor effects. To confirm the immune‐activating effect of CuPB/Mt, the expression of NLRP3 pathway‐related proteins was assessed in vivo via immunofluorescence staining. As expected, the expression of cleaved caspase‐1 (downstream of pyroptosis) was significantly enhanced in the CuPB/Mt group compared with that in the other groups, indicating the occurrence of intense pyroptosis in the tumors. In addition, the expression of NLRP3 was greatly upregulated in the CuPB/Mt group (Figure 6d), indicating that CuPB/Mt could substantially activate inflammasomes and induce NLRP3‐dependent pyroptosis in vivo, finally evoking innate and adaptive immune responses.

### 2.9 In Vivo Anti‐Tumor Immune Responses

Furthermore, the in vivo anti‐tumor immune responses induced by CuPB/Mt were investigated. Since DCs play a key role in antigen presentation and subsequent T‐cell activation, the maturation of tumor‐infiltrating DCs (CD80^+^/CD86^+^ populations) was quantified using flow cytometry. As shown in Figure 6e and Figure S23a, CuPB/Mt treatment induced the highest DC maturation rate, which was 3.08‐fold higher than that in the PBS group. Meanwhile, the proportions of CD4^+^ and CD8^+^ T cells in the CuPB/Mt group were increased by 2.47‐ and 4.26‐fold, respectively, compared with those in the PBS group, indicating enhanced T‐cell infiltration within the tumor microenvironment (Figure 6f,g and Figure S23b,c). Concurrently, the secretion of pro‐inflammatory cytokines, including TNF‐𝛼, IFN‐γ, IL‐6 and IL‐1𝛽, was significantly elevated in the CuPB/Mt group (Figure 6h–j and Figure S24). These results suggest that CuPB/Mt effectively promoted in vivo immune activation, consistent with the induction of pyroptotic inflammatory signaling.

Notably, the Mt group also showed a mild immune‐activation tendency compared with the PBS group, which may be related to the intrinsic immunoadjuvant‐like properties of clay minerals. Nevertheless, Mt alone did not induce obvious tumor suppression, whereas CuPB/Mt triggered much stronger DC maturation, T‐cell infiltration, and cytokine secretion. Therefore, the immune activation induced by CuPB/Mt likely resulted from the cooperation between Mt‐mediated immunoadjuvant‐like effects and catalytic ROS‐mediated pyroptosis.

## 3 Conclusion

In summary, we successfully constructed a self‐amplifying copper‐based Bio‐Clayzyme (CuPB/Mt) for breast tumor therapy by integrating mineral‐mediated catalytic enhancement with endogenous perturbation of copper homeostasis. The introduction of Mt served dual functions in regulating both the catalytic interface and cellular interactions of CuPB. At the CuPB/Mt interface, Mt facilitated charge transfer and electron injection toward Cu sites, resulting in an upward shift of the Cu d‐band center. DFT calculations further revealed that this electronic modulation strengthened the adsorption of key reaction intermediates in the POD‐like catalytic pathway and lowered the energy barrier of the rate‐determining step, thereby accelerating the catalytic process. Consequently, CuPB/Mt exhibited significantly enhanced POD‐, GSHOx‐, and NOx‐like activities compared with CuPB, enabling efficient intracellular GSH depletion while suppressing its regeneration and thereby amplifying oxidative stress through cascade catalytic reactions. Meanwhile, the intrinsic cellular affinity of Mt promoted the internalization and intracellular enrichment of CuPB/Mt, facilitating proximity‐enhanced utilization of short‐lived ROS. The resulting oxidative stress induced mitochondrial dysfunction and ATP depletion, which subsequently caused the reduction of ATP7A expression and an increase of intracellular copper retention. Elevated intracellular copper may, in turn, further facilitate Cu‐based redox cycling and ROS generation, supporting a self‐amplifying feedback process among copper accumulation, oxidative stress, mitochondrial dysfunction, ATP depletion, and ATP7A downregulation. This sustained oxidative perturbation activated the NLRP3/cleaved caspase‐1/GSDMD pathway, promoted membrane permeabilization and inflammatory mediator release, and ultimately elicited robust immunogenic pyroptosis. In vivo, CuPB/Mt effectively suppressed tumor growth while promoting DC maturation, T‐cell infiltration, and pro‐inflammatory cytokine secretion, reflecting the cooperative contributions of Mt‐mediated immunoadjuvant‐like effects and catalytic ROS‐driven pyroptosis. Overall, this work establishes an ATP7A‐mediated endogenous regulatory strategy that couples mineral‐enabled catalytic enhancement with copper‐homeostasis perturbation, providing a promising framework for self‐amplified catalytic therapy and pyroptosis‐based tumor immunotherapy.

## Author Contributions

**Ying Chen** and **Huaming Yang** conceived the idea and designed the project. **Weimin Xie** performed the experiments, characterizations, and mechanistic analyses. **Yan Li** and **Guangyao Li** assisted the animal experiments. Weimin Xie, Ying Chen and Huaming Yang discussed the results. Weimin Xie wrote initial drafts of the manuscript. Ying Chen and Huaming Yang revised the final manuscript. All authors discussed the results and reviewed the manuscript.

## Conflicts of Interest

The authors declare no conflicts of interest.

## Supporting information




**Supporting File**: advs77973‐sup‐0001‐SuppMat.docx.

## Data Availability

The data that support the findings of this study are available from the corresponding author upon reasonable request.
