# Supplementary material for: An ATP7A‐Mediated Endogenous Regulation Bio‐Clay Enzyme Enables Self‐Amplified Copper Accumulation for Immunogenic Pyrop
Source: Adv Sci (Weinh). 2026 Oct 11:e77973. Online ahead of print. doi: 10.1002/advs.77973 (PMC13654860; doi:10.1002/advs.77973)
Supplement: Supplementary file 1 [file ADVS-9999-e77973-s001.docx]

Supporting Information

**An ATP7A-mediated Endogenous Regulation Bio-clay Enzyme Enables Self-Amplified Copper Accumulation for Immunogenic Pyroptosis**

Weimin Xie ^1^, Yan Li ^1,2,3^, Guangyao Li ^1,2,3^, Ying Chen ^1,2,3*^, Huaming Yang ^1,2,3*^

^1^ Engineering Research Center of Nano-Geomaterials of Ministry of Education

China University of Geosciences, Wuhan 430074, China

^2^ Faculty of Materials Science and Chemistry

China University of Geosciences, Wuhan 430074, China

^3^ Laboratory of Advanced Mineral Materials

China University of Geosciences, Wuhan 430074, China

*Corresponding authors: chenying2021@cug.edu.cn (Y. Chen), hm.yang@cug.edu.cn (H. Yang)

**This PDF file includes:**

Experimental Section

Supplementary Figures 1 to 24

Supplementary Tables 1 to 3

**Supplementary Figures**

**Figure S1.** The corresponding element mapping images of CuPB.

**Figure S2.** XPS spectra of Mt, CuPB and CuPB/Mt.

**Figure S3.** (a) Thermogravimetric analysis curves of Mt, CuPB and CuPB/Mt, (b) The percentage of CuPB and Mt in CuPB/Mt.

**Figure S4.** Hydrodynamic sizes of Mt, CuPB and CuPB/Mt.

**Figure S5.** Zeta potential of Mt, CuPB and CuPB/Mt.

**Figure S6.** XRD patterns of Mt, CuPB and CuPB/Mt.

**Figure S7.** FTIR spectra of Mt, CuPB and CuPB/Mt.

**Figure S8.** FT-EXAFS spectra of CuPB, CuPB/Mt and the reference samples.

**Figure S9.** Images of color changes in the reaction solutions after treatment with TMB and H_2_O_2_.

**Figure S10.** Images of color changes in the reaction solutions after treatment with GSH and DTNB.

**Figure S11.** The optimized spatial configurations of Mt.

**Figure S12.** CLSM images of endocytosis after different treatments (scale bar: 50 µm).

**Figure S13.** Calculation of the JC-1 monomer/JC-1 aggregate ratio after different treatments.

**Figure S14.** WB analysis of copper-homeostasis-related proteins, including ATP7B, CTR1, and ATOX1, in 4T1 cells after different treatments.

**Figure S15.** Total cellular protein content after different treatments.

**Figure S16.** WB analysis of ATP7A expression in parental 4T1 cells (left) and ATP7A-upregulated 4T1 cells (right).

**Figure S17.** Bright-field images of 4T1 cells after different treatments (scale bar: 50 μm).

**Figure S18.** Body weight change of 4T1 tumor-bearing mice during the treatment process.

**Figure S19.** Isolated tumor images at the end of the treatment.

**Figure S20.** H&E staining images of major organs, including heart, liver, spleen, lung, and kidney (scale bar: 200 μm).

**Figure S21.** The blood routine analysis including the levels of WBC, RBC, HCT, HGB, MCHC, MCH, MCV and PLT.

**Figure S22.** The blood biochemistry analysis including the levels of ALT, AST, ALB, TP, UREA and Cr.

**Figure S23.** Representative flow cytometry plots indicating the proportions of (a) CD80^+^CD86^+^ DCs, (b) CD3^+^CD4^+^ T cells, and (c) CD3^+^CD8^+^ T cells in the tumors.

**Figure S24.** The secretion levels of IL-1*β* in serum of mice.

**Supplementary Tables**

**Table S1.** EXAFS fitting parameters at the Cu K–edge for various samples.

**Table S2.** Comparison of kinetics for CuPB and CuPB/Mt.

**Table S3.** Reaction free energy of intermediate species on CuPB and CuPB/Mt.

**Experimental Section**

**Materials.** Medical montmorillonite (Mt), potassium ferrocyanide (K_4_[Fe(CN)_6_]), 3,3′,5,5′-tetramethylbenzidine (TMB) were purchased from Macklin Reagent Co., ltd. (China). Copper chloride dihydrate (CuCl_2_·2H_2_O), citric acid, and glutathione (GSH) were obtained from Aladdin Reagent Co., Ltd. (China). Hydrogen peroxide (H_2_O_2_, 30%) was purchased from Sinopharm Chemical Reagent Co., Ltd. DTNB (98%) was purchased from Yuanye Bio-Technology Co., Ltd. (China). Nicotinamide adenine dinucleotide (NADH), 2′7′-dichlorofluorescin diacetate (DCFH-DA), Hoechst 33342, Calcein-AM, propidium iodide (PI), Annexin V-FITC/PI apoptosis detection kit, mitochondrial membrane potential assay kit with JC-1, NAD^+^/NADH assay kit, GSH and GSSG assay kit, ATP assay kit and LDH cytotoxicity assay kit were purchased from Beyotime Institute of Biotechnology (China). Enzyme-linked immunosorbent assay (ELISA) kits for IL-1*β*, TNF-*α*, IFN-*γ* and IL-6 were purchased from Bioswamp®. Recombinant murine IL-4 and recombinant murine GM-CSF were purchased from Novoprotein Technology Co., Ltd. (China). Anti-mouse antibodies: CD80-PE, CD86-APC, CD11c-FITC, CD3-FITC, CD4-PE and CD8-APC were obtained from Thermo Fisher (USA). All reagents were used as received without further purification.

**Preparation and characterization** **of CuPB/Mt.** 0.1 mmol of CuCl_2_·2H_2_O was dissolved in 50 mL of an aqueous solution containing 40 mg of PDDA and 50 mg of Mt with stirring for 6 h, followed by the addition of 250 mg of citric acid. The obtained solution was heated to 60 °C. Subsequently, 50 mL of an aqueous solution containing 0.1 mmol of K_4_[Fe(CN)_6_] and the same amount of citric acid was added dropwise under vigorous stirring. The resulting mixture was stirred vigorously at 60 °C for 2 h and at room temperature for 12 h. Finally, the CuPB/Mt was obtained after washing three times with deionized water. Transmission electron microscopy (TEM) was performed using a JEOL JEM-F200 microscope. X-ray photoelectron spectroscopy (XPS) was performed using a Thermo Scientific K-Alpha instrument. X-ray diffraction (XRD) patterns were recorded using a Bruker D8 ADVANCE diffractometer with Cu Kα-radiation (λ = 0.154 nm). Fourier transform infrared (FTIR) spectra were measured by Thermo Fisher Nicolet iS50. X-ray absorption fine structure (XAFS) measurements were performed at the XAS Beamline at the Australian Synchrotron (ANSTO) in Melbourne, Australia.

**Detection of multiple enzymatic activities.**

POD-like: the POD-like activity was investigated in the presence of TMB and different concentrations of H_2_O_2_ solution. The absorbance of oxTMB was recorded by a microplate reader at 652 nm (ε=39000 M^-1^cm^-1^).

GSHOx-like: the GSHOx-like activity was measured in the presence of DTNB and different concentrations of GSH. The absorbance was monitored by a microplate reader at 412 nm (ε = 13600 M^-1^ cm^-1^).

NOx-like: the NOx-like activity was performed using different concentration of NADH. The absorbance was recorded by a microplate reader at 340 nm (ε = 6220 M^-1^cm^-1^).

The kinetic parameters of the above three enzymatic activities are calculated by the following equation: 1/$\nu$= *K*m/$\nu$_max_(1/[*S*]+1/*K*m), where $\nu$_max_ represents the maximum reaction velocity, [*S*] represents the substrate concentration, $\nu$ represents the initial reaction velocity, and *K*m is the Michaelis constant. The catalytic constant (*K*cat) was acquired by the following equation: *K*cat =$\nu$_max_/[*E*]**,** where *K*cat is the rate constant. [*E*] represents the nanozyme concentration (M). In this research, [*E*] refers to the molar concentration of the Cu catalytic component used in each reaction system.

**DFT calculations.** The density functional theory computations were performed using the Vienna Ab initio Simulation Package (VASP) using the projector augmented wave (PAW) method. For the interfacial model, the CuPB (200) surface and the Mt (001) basal plane were selected to construct the CuPB/Mt interface. The CuPB (200) surface was used as a representative exposed surface of the CuPB structure, and the Mt (001) plane was chosen as the dominant basal surface of layered Mt. The exchange-correlation potential was represented by the Perdew–Burke–Ernzerhof (PBE) functional within the generalized gradient approximation (GGA). The vacuum layer was set to 15 Å. The cutoff energy for the plane-wave-basis expansion was set to be 450 eV, and a 2×1×1 *k*-point sampling grid was applied. The structures were relaxed until the convergence tolerances of energy and force were less than 1.0×10^-5^ eV and 3.0×10^-2^ eV/Å. Moreover, the DFT+ U method was used to describe the strong correlation of *d* electrons. The adsorption energy (*E*_ad_) of H_2_O_2_ was calculated by:

*E*_ad_ = *E*(total) − *E*(slab) − *E*(H_2_O_2_)

Where *E*(total) is the total energy of the system after H_2_O_2_ adsorption on the substrate surface, *E*(slab) is the energy of the substrate surface, *E*(H_2_O_2_) is the energy of the H_2_O_2_ molecule.

The Gibbs free energy change (Δ*G*) of the elementary step was calculated by:

∆*G* = ∆*E* + ∆*E*_ZPE_ − *T*∆*S*

Where ∆*E* is the total energy difference, Δ*E*_ZPE_ is the change in the zero-point energy and the entropy, *T* is the temperature (*T* = 298.15 K), and Δ*S* is the entropy change.

**Cell culture.** 4T1 tumor cells were obtained from China Center for Type Culture Collection (CCTCC) and incubated in RPMI 1640 medium containing 10% fetal bovine serum (FBS) and 1% penicillin-streptomycin in an incubator at 37 ℃ with 5% CO_2_.

**Cytotoxicity evaluation.** The 4T1 cells were seeded in 96-well plates (1 × 10^4^/well) for 24 h. Then, the cells were co-cultured with PBS, Mt, CuPB and CuPB/Mt with different concentrations for another 24 h. Subsequently, the cytotoxicity was detected using a CCK-8 assay (Biosharp Co., Ltd).

**Annexin V-FITC/PI staining assay**. The 4T1 cells were seeded in 6-well plates (1 × 10^5^/well) for 24 h. Then, the cells were respectively incubated with PBS, Mt, CuPB and CuPB/Mt. Subsequently, the cell suspension was collected and stained with an Annexin V-FITC/PI apoptosis detection kit and analyzed by flow cytometry.

**Living/Dead Cell Staining Assay.** The 4T1 cells were seeded in 6-well plates (2 × 10^5^/well) for 24 h. Subsequently, the cells were incubated with PBS, Mt, CuPB and CuPB/Mt. The cells were then stained with Calcein AM/PI and observed by CLSM.

**Evaluation of intracellular homeostasis.** The 4T1 cells were seeded in 6-well plates (1 × 10^5^/well) for 24 h. Then, the cells were respectively incubated with PBS, Mt, CuPB and CuPB/Mt. Subsequently, the intracellular and extracellular ATP levels were measured by an enhanced ATP assay kit. The intracellular NADH and GSH levels were detected by using a NAD^+^/NADH assay kit and a GSH and GSSG assay kit according to the manufacturer’s instructions.

**Intracellular ROS detection.** The 4T1 cells were incubated in 35-mm glass-bottom cell culture dishes for 24 h. After that, the cells were incubated with PBS, Mt, CuPB and CuPB/Mt. Subsequently, the cells were stained with DCFH-DA and then detected using CLSM and flow cytometry.

**Mitochondrial membrane potential detection**. The 4T1 cells were seeded in 6-well plates (1 × 10^5^/well) for 24 h. Then, the cells were incubated with PBS, Mt, CuPB and CuPB/Mt. After that, the mitochondrial membrane potential was investigated via JC-1 staining according to the manufacturer’s protocol and photographed by CLSM.

**Intracellular copper detection.** The 4T1 cells were seeded in 6-well plates (1 × 10^5^/well) for 24 h. Then, the cells were incubated with PBS, Mt, CuPB and CuPB/Mt. Subsequently, the intracellular copper content was detected using a copper colorimetric assay kit (Elabscience Biotechnology Co. Ltd.).

**Detection of pyroptosis-related protein expression.** The 4T1 cells were seeded in 6-well plates (1 × 10^5^/well) for 24 h. Then, the cells were incubated with PBS, Mt, CuPB and CuPB/Mt. After treatments, the cells were washed with PBS and lysed with cell lysis buffer on ice. Then the lysates were analyzed through western blot to detect the protein expression levels of NLRP3, cleaved caspase-1, GSDMD-N, with *β*-actin serving as the reference protein.

**LDH release detection:** The 4T1 cells were inoculated in 96-well plates (1×10^4^ cells per well) for 24 h. Then, the cells were respectively incubated with PBS, Mt, CuPB and CuPB/Mt. Subsequently, LDH release was evaluated using an LDH cytotoxicity assay kit following the manufacturer’s protocols.

**BMDC maturation.** Bone marrow dendritic cells (BMDCs) were isolated from 8-week-old BALB/c mice and incubated in RPMI 1640 complete medium containing IL-4 (10 ng/mL) and GM-CSF (20 ng/mL) for 7 days. On day 8, the above immature DCs were placed in the lower chamber of the Transwell system, while differently treated 4T1 cells were seeded in the upper chamber for another 24 h. Afterwards, the DCs were stained with CD11c, CD80 and CD86, followed by analysis with flow cytometry. In addition, the supernatants were also collected to detect the secretion of cytokines by ELISA kit.

**Animal models.** All animal procedures were conducted in compliance with the National Institute of Health Guidelines, approved by the Animal Ethics Committee of Wuhan Myhalic Biotechnology Co., Ltd. (Ethics Committee number: HLK-20241106-001).

***In vivo* anti-tumor effect.** 4T1 tumor models were established by subcutaneously injecting 4T1 cells (1×10^6^ cells/mouse) into the back of female BALB/c mice. When the tumors grew to about 80 mm^3^, 4T1 tumor-bearing mice were randomly assigned to four groups and intravenously injected with PBS, Mt, CuPB and CuPB/Mt (at CuPB concentration of 600 μg/mL, 100 μL/mouse) on days 0, 2, and 4. The tumor sizes and body weights were monitored during the treatments. Tumor volumes V= width^2^ × length/2. On day 11, a portion of tumors were collected for DC maturation and detection of intratumoral immune responses through flow cytometry. Additionally, the serum of mice was also collected to evaluate the levels of immune-related cytokines (TNF-*α*, IFN-*γ*, IL-6 and IL-1*β*) using ELISA. On day 20, all mice were sacrificed and the tumors were harvested for hematoxylin-eosin (H&E) staining and immunofluorescence assays, the main organs were obtained for H&E staining, and the blood samples were collected for blood biochemistry and blood routine analysis.

**Statistical analysis.** All statistical analyses were conducted with SPSS software (version 19.0, IBM, USA). Statistical data were represented as the mean ± standard deviation (SD) from at least three independent experiments. Data comparisons were performed using one-way analysis of variance (ANOVA) and then Tukey’s honestly significant difference (Tukey’s HSD) post-hoc test. Statistical significance was determined based on p-values, with significance levels indicated as (*) for p<0.05, (**) for p<0.01, (***) for p<0.001.

**Figure S1.** The corresponding element mapping images of CuPB.

**Figure S2.** XPS spectra of Mt, CuPB and CuPB/Mt.

**Figure S3.** (a) Thermogravimetric analysis curves of Mt, CuPB and CuPB/Mt, (b) The percentage of CuPB and Mt in CuPB/Mt.

**Figure S4.** Hydrodynamic sizes of Mt, CuPB and CuPB/Mt.

**Figure S5.** Zeta potential of Mt, CuPB and CuPB/Mt.

**Figure S6.** XRD patterns of Mt, CuPB and CuPB/Mt.

**Figure S7.** FTIR spectra of Mt, CuPB and CuPB/Mt.

**Figure S8.** FT-EXAFS spectra of CuPB, CuPB/Mt and the reference samples.

**Figure S9.** Images of color changes in the reaction solutions after treatment with TMB and H_2_O_2_.

**Figure S10.** Images of color changes in the reaction solutions after treatment with GSH and DTNB.

**Figure S11.** The optimized spatial configurations of Mt.

**Figure S12.** CLSM images of endocytosis after different treatments (scale bar: 50 µm).

**Figure S13.** Calculation of the JC-1 monomer/JC-1 aggregate ratio after different treatments.

**Figure S14.** WB analysis of copper-homeostasis-related proteins, including ATP7B, CTR1, and ATOX1, in 4T1 cells after different treatments.

**Figure S15.** Total cellular protein content after different treatments.

**Figure S16.** WB analysis of ATP7A expression in parental 4T1 cells (left) and ATP7A-upregulated 4T1 cells (right).

**Figure S17.** Bright-field images of 4T1 cells after different treatments (scale bar: 50 μm).

**Figure S18.** Body weight change of 4T1 tumor-bearing mice during the treatment process.

**Figure S19.** Isolated tumor images at the end of the treatment.

**Figure S20.** H&E staining images of major organs, including heart, liver, spleen, lung, and kidney (scale bar: 200 μm).

**Figure S21.** The blood routine analysis including the levels of WBC, RBC, HCT, HGB, MCHC, MCH, MCV and PLT.

**Figure S22.** The blood biochemistry analysis including the levels of ALT, AST, ALB, TP, UREA and Cr.

**Figure S23.** Representative flow cytometry plots indicating the proportions of (a) CD80^+^CD86^+^ DCs, (b) CD3^+^CD4^+^ T cells, and (c) CD3^+^CD8^+^ T cells in the tumors.

**Figure S24.** The secretion levels of IL-1*β* in serum of mice.

**Table S1.** EXAFS fitting parameters at the Cu K–edge for various samples.

| Sample | Shell | *CN* | *R* (Å) | *σ*^2^ (Å^2^) | Δ*E*_0_ (eV) | *R* factor |
| --- | --- | --- | --- | --- | --- | --- |
| Cu foil | Cu-Cu | 12* | 2.54±0.01 | 0.0086±0.0003 | 4.2±0.2 | 0.0015 |
| Cu_2_O | Cu-O | 2* | 1.85±0.01 | 0.0027±0.0010 | 8.0±0.5 | 0.0128 |
|  | Cu-Cu | 12* | 3.02±0.01 | 0.0235±0.0014 | 6.3±0.5 |  |
|  | Cu-O | 6* | 3.46±0.01 |  |  |  |
| CuO | Cu-O | 4.0±0.3 | 1.96±0.01 | 0.0060±0.0011 | 7.4±0.4 | 0.0074 |
|  | Cu-Cu | 2.3±0.5 | 2.85±0.01 | 0.0083±0.0019 | -2.5±0.9 |  |
|  | Cu-Cu | 2.0±0.5 | 3.42±0.01 |  |  |  |
| CuPB | Cu-C | 5.1±0.6 | 1.98±0.01 | 0.0059±0.0014 | 2.9±0.8 | 0.0080 |
|  | Cu-N | 7.6±1.5 | 3.18±0.01 | 0.0058±0.0027 | 5.4±0.7 |  |
| CuPB/Mt | Cu-O | 2.3±0.4 | 1.91±0.01 | 0.0042±0.0008 | 4.6±0.5 | 0.0073 |
|  | Cu-C | 2.3±0.8 | 2.01±0.01 |  |  |  |
|  | Cu-N | 8.4±0.8 | 3.17±0.01 |  |  |  |

*CN*: coordination number; *R*, bond distance; *σ*^2^, Debye-Waller factor; *ΔE*_0_, inner potential correction; *R* factor: goodness of the fit. S_0_^2^ was fixed to 0.860.

**Table S2.** Comparison of kinetics for CuPB and CuPB/Mt.

| Sample | *E* (M) | Substrates | *K*m (mM) | $\nu$_max_ (M s^-1^) | *K*cat (s^-1^) | *K*cat/*K*m (mM^-1^s^-1^) |
| --- | --- | --- | --- | --- | --- | --- |
| CuPB | 2.29×10^-4^ | H_2_O_2_ | 47.30 | 3.90×10^-7^ | 1.70×10^-3^ | 3.60×10^-5^ |
|  |  | GSH | 1.48 | 8.71×10^-9^ | 3.80×10^-5^ | 2.57×10^-5^ |
|  |  | NADH | 0.22 | 1.42×10^-9^ | 6.20×10^-6^ | 2.82×10^-5^ |
| CuPB/Mt | 2.18×10^-4^ | H_2_O_2_ | 43.68 | 5.75×10^-7^ | 2.65×10^-3^ | 6.07×10^-5^ |
|  |  | GSH | 1.52 | 1.40×10^-8^ | 6.42×10^-5^ | 4.23×10^-5^ |
|  |  | NADH | 0.43 | 3.41×10^-9^ | 1.56×10^-5^ | 3.64×10^-5^ |

**Table S3.** Reaction free energy of intermediate species on CuPB and CuPB/Mt

| Free energy  (eV)  Sample | H_2_O_2_* | 2OH* | OH*+·OH | H_2_O* |
| --- | --- | --- | --- | --- |
| CuPB | -0.02996 | 0.18226 | -0.40544 | -2.91109 |
| CuPB/Mt | -0.06221 | -0.03008 | -0.81032 | -3.00956 |
